# A New Design of a Thin-Film Thermoelectric Device Based on Multilayer-Structure Module

**DOI:** 10.3390/nano10050990

**Published:** 2020-05-21

**Authors:** Tianbao Chen, Zhuanghao Zheng, Guangxing Liang, Ping Fan

**Affiliations:** Shenzhen Key Laboratory of Advanced Thin Films and Applications, College of Physics and Optoelectronic Engineering, Shenzhen University, Shenzhen 518060, China; chentianbao@caihuang.com (T.C.); zhengzh@szu.edu.cn (Z.Z.); lgx@szu.edu.cn (G.L.)

**Keywords:** thermoelectric, thin film, multilayer, device

## Abstract

In this work, a novel multilayer structure thin-film thermoelectric device is proposed for preparing a high performance generator. The result shows that the output voltage of the three-layer thin-film device has a linear increasing trend with the increasing temperature difference. Additionally, the device was also tested as a laser power measurement and displays that it has good sensitivity. Moreover, we also fabricated the multilayer device based on the present three-layer structure. It improves upon the similar output prosperities, confirming that the present multilayer structure thin-film thermoelectric device can be considered for preparing high performance micro-self-powered sources and sensors.

## 1. Introduction

Thermoelectric (TE) technology can convert heat into electricity directly and it has many advantages, such as being environmental friendly, having low-cost operation and good reliability, and so on [1]. So, this technology has attracted much attention due to its many advantages and wide applications [2]. Recently, with the development of micro-electronics and an increasing demand for sustainable energy harvesting, high power density TE devices that are micro-sized and light-weight have become the subject of rapidly growing interest [3,4]. Compared with traditional brittle and rigid TE devices, a micro TE device is essential to obtain low temperatures such as that of the human body or flexible electronic devices, thus, minimizing heat loss and achieving highly efficient energy conversion [5]. Additionally, the integration of a TE device into the micro-system can act as the self-powered source, cooler, or sensor, and has significant potential for practical application. Thus, motivated by these intriguing prospects, considerable effort has been devoted to exploring micro-TE devices in the past decade [6,7,8,9,10]. Among many of the reports, micro-TE devices fabricated based on thin-film technology has been widely studied due to the free-standing thin-film thermoelectric materials which are always preferred to obtain optimum device configurations. It is because they can be easily transferred onto any substrate, enabling remarkable improvements in efficiency by reducing thermal energy losses [11]. Moreover, low-dimensional thin-film materials can achieve a very high Dimensionless thermoelectric figure of merit (ZT) value, leading to a high efficiency TE device [12]. For instance, Venkatasubramanian et al. [13] achieved a very high ZT value material of about 2.4 by using a superlattice structure. Then, Chowdhury et al. [14] made a thin-film TE device with the same concept, and fabricated it into state-of-the-art electronic packages with cooling to a high (~1300 Wcm^−2^) heat flux. Tian et al. [15] prepared an organic superlattices generator with very high power density of 2.5 Wm^−2^ which is almost one hundred times higher than the others. 

**Figure 1:**
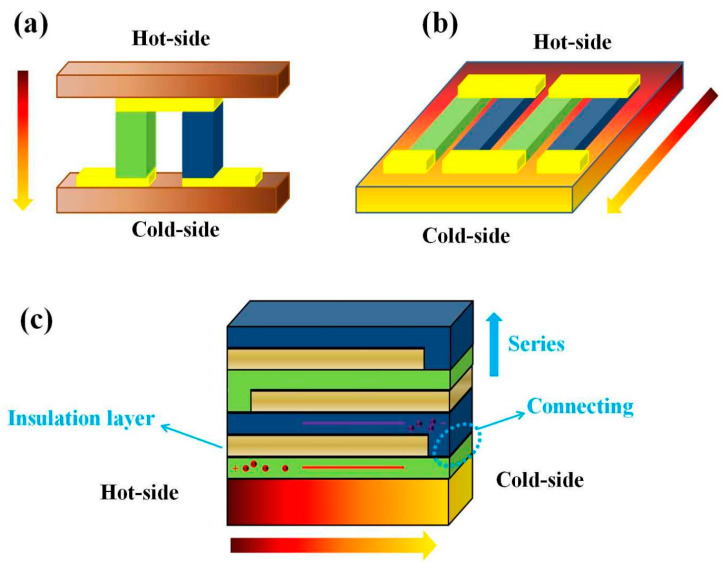
The structure schematic of the thin-film thermoelectric (TE) device (TFTED): (**a**) across-plane, (**b**) in-plane, and (**c**) multilayer structure.

However, there are some important challenges, including the high preparation cost and poor thermal stability that remain for thin-film TE devices (TFTEDs) used for practical applications. Especially, the structural design of the TFTED needs to be improved in order to enhance its performance. Generally, there are two main structures for TFTED as shown in the Figure 1a of the support information. One usual design is the heat flux perpendicular to the plane of the TE films, which is similar to the bulk device [10]. Although this traditional design allows convenient connection between the TE legs, this across-plane device is unable to obtain a high temperature difference as restricted by the thin-film thickness. It will limit the output power of TE device. Recently, many reports deal with the other structure of the TFTED [15], such as the in-plane heat transfer structure in Figure 1b, which can create a large temperature gradient due to the long TE legs. Unfortunately, the electrical resistance is the issue when the current flows in the plane of the thin-film device. For these reasons, a new structural design of TFTEDs with a simple fabricating process and excellent performance has been considered as an essential approach to achieving the requirements of practical application.

In this paper, a novel multilayer scheme is proposed for TFTEDs with the heat flux and electrical current parallel to the thin-film surface. Different to the traditional in-plane device, all the thin films of the multilayer TFTED are set layer by layer with the growth direction across the thin-film surface as shown in Figure 1c. For details, a P-type TE thin-film layer, an insulating thin-film layer, and an N-type TE thin-film layer are deposited in sequence onto a substrate to form a three-layer structure. By using intentional sheltering, one end of the deposited TE thin films are connected with each other to form the PN junction. Then, multiple three-layer PN junctions in series are available, with an insulating thin-film layer between every three-layer PN junctions. This simply fabrication process allows the TFTED to possess huge amount of PN junctions in a small volume size by controlling the layers numbers. The proposed multilayer structure enables a high integration of the TE elements. It can enable the device to acquire very high output voltage by combining over hundreds of TE PN-modules in a small scale range. Thus, it provides a possibility to simplify the manufacturing process and avoid using the large length-width area.

## 2. Experimental Details and Results

**Figure 2:**
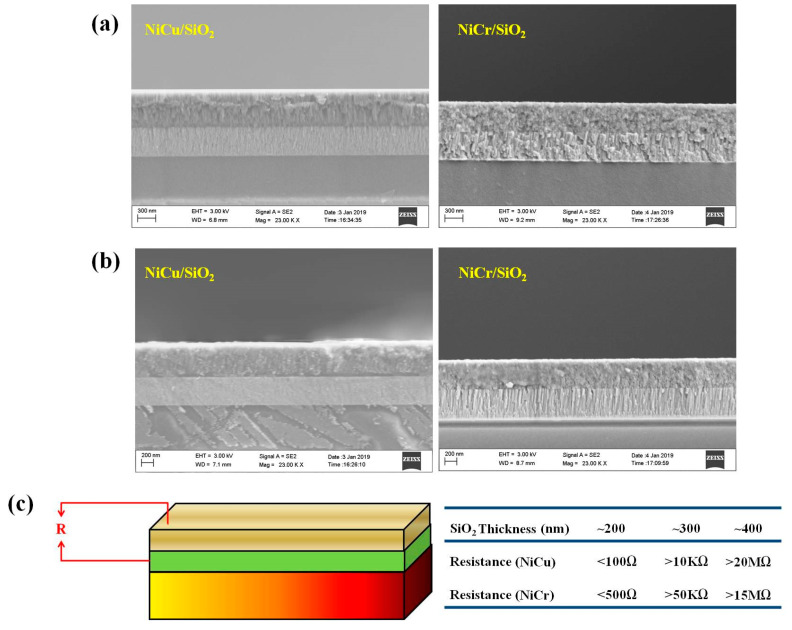
(**a**) The cross-section images of NiCu/SiO_2_ and NiCr/SiO_2_ thin films; (**b**) The cross-section images of NiCu/SiO_2_ and NiCr/SiO_2_ thin films after heating; and (**c**) The resistance between the TE thin films and SiO_2._

To demonstrate the feasibility of this new design, we used the traditional NiCu and NiCr materials, which have been widely used in commercial TE sensor applications, as the p-type and n-type elements. The SiO_2_ was used as the insulted layer in this paper. First of all, all of the thin films were synthesized by the magnetron sputtering deposition method and characterized. We chose the BK7 glass with a thickness of 2 mm and a surface roughness of <10 nm as the substrate. The Seebeck coefficient and electrical conductivity of the thin films were measured by the simultaneous determination of Seebeck coefficient and electrical conductivity system (SBA458). The detail preparing parameters and properties of the thin films are listed in Table 1. As shown in Table 1, the Seebeck coefficient is negative and the absolute value is 28 μV K^−1^ for the NiCu thin film, while a positive value of 15 μV K^−1^ is observed for NiCr thin film. Therefore, a prospective thermo-power with one pair of PN leg of the device is about 43 μV K^−1^. Both the NiCr and NiCu thin film have very high electrical conductivity, thus leading to low-contact resistance after connecting. The prepared SiO_2_ layer has very low-electrical conductivity. Then, the double-layer films as “NiCr/SiO_2_” and “NiCu/SiO_2_” were prepared. The cross-sections of those thin films were examined and shown in Figure 2a by using a scanning electron microscopy (Zeiss supra 55). It can be found that the prepared double-layer thin films have good adhesion characteristics and few interface defects between the layers. Additionally, the cross-sections of these thin films after being heated to 400 K, in Figure 2b, display that all the thin films have few changes. It indicates the good thermal stability of the thin films we fabricated. At the same time, we also measure the resistance between the TE thin films and insulating layer shown in Figure 2c. The insert table in Figure 2c shows the influence of the SiO_2_ thickness on the resistance. It displays that the resistance increases from about 100 Ω to over 20 MΩ when the thickness increases from ~200 nm to ~400 nm, which is sufficient for the blocked level to ensure the electrons and holes can only migrate according to the temperature gradient in the TE thin films.

**Figure 3:**
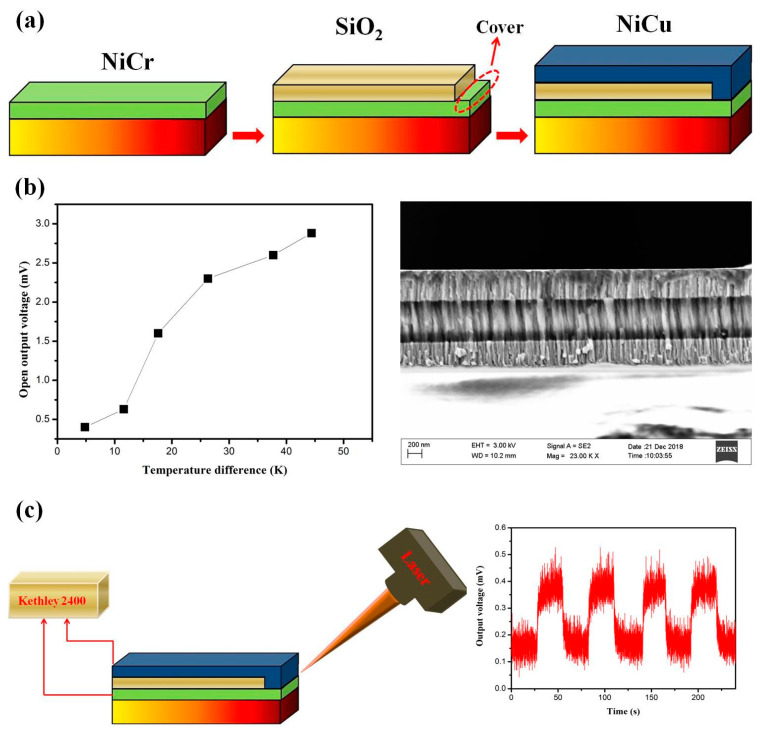
(**a**) The preparing process of the three-layer TFTED; (**b**) The open output voltage of the TFTED as function of temperature difference and the cross-section image; and (**c**) An illustration of the laser power measurement application by using TFTED and the continuous response testing.

The TFTED with three-layer structure as “NiCr–SiO_2_–NiCu” was fabricated and a schematic representation of the process is shown in Figure 3a. At first, the p-type thin film “leg” was deposited on the glass substrate and the thickness was around 400 nm. Then, the SiO_2_ layer was deposited onto the P-type thin film with a mask which covered one side of the TE leg, leaving a connecting end. After that, the N-type thin film “leg” with the similar thickness as the P-type thin film was then deposited onto the SiO_2_ layer, and the TE thin films were connected by the reserved side, thus forming the PN junction. The output voltages and short-circuit currents of the device were measured in normal atmosphere with a Keithley 2400 as a function of the temperature difference between hot and cold sides. Figure 3b shows the open output voltage (V_o_) of the TFTED with a single PN junction as a function of temperature difference, ΔT. The imposed temperature gradient is parallel to the length of the TFTED leg. The result reveals that the V_o_ of the device increases linearly with the increasing temperature difference. The linear fit of the experimental data yields a Seebeck coefficient of about 45 μV K^−1^, which agrees well with the prospective value of the PN junction of about 43 μV K^−1^. The cross-sectional image measured by SEM was insert in Figure 3b, showing that all of the thin film layers has good adhesion characteristics and few interface defects between the layers. Besides, no obvious contact defect can be seen from the connecting side of the P-type and N-type layer. Furthermore, a TE device is favorable for highly sensitive detector applications. Therefore, a laser power measurement application using this multilayer device was also tested as shown in Figure 3c. The Kethley 2400 and a laser which used the continuous monitoring mode with an interval time of 0.05 s were employed. The continuous response of the TFTED was measured by the laser beam with respective 50 s interval irradiating the connecting region of the device as “on” and “off”, and the irradiation time was 25 s. The output signal as the function of the testing time is also shown in Figure 3c. It can be seen that the voltage increases rapidly in response to the ‘‘on” state and has the maximum output voltage about 0.4 mV, suggesting that the thermal gradient is about 10 K (0.4 mv/0.45 uvK − 1 = 8.89 K). Especially, the rise time of reaching the maximum voltage for each laser beam is about 2–3 s and this TFTED had stable output voltage when the laser beam continued irradiating. This response time is shorter than the commercial laser power detector which needs over 5 s to achieve a stable value. Additionally, we also measured the response time of traditional bulk thermocouples, fabricated with NiCu and NiCr by using the same method. It displays that the response time is about 50% higher than that of our prepared thin film device, indicating the TFTED can be considered for using as a fast response sensor. 

**Figure 4:**
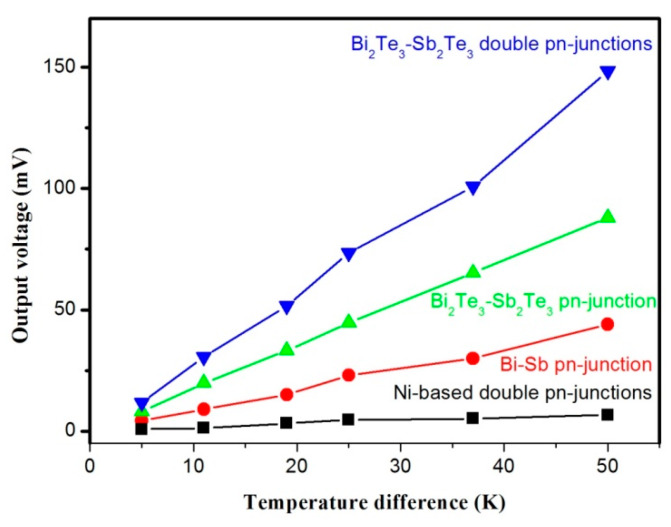
The open output voltage of the TFTED with various materials as function of temperature difference.

In order to further investigate the reliability of the multilayer structure, the TFTED was fabricated by using different TE materials. The N-type Bi and P-type Sb, which have higher TE properties and very weak temperature dependence compared to the traditional Ni-based materials, were used for fabricating the TFTED. In addition, the N-type Bi_2_Te_3_ and P-type Sb_2_Te_3_ were also employed due to their good thermoelectric performance at room temperature. The room-temperature TE properties of Bi, Sb, Bi_2_Te_3_, and Sb_2_Te_3_ prepared by magnetron sputtering disposition are shown in Table 2. Besides one PN junction, the Ni-based and Bi_2_Te_3_/Sb_2_Te_3_ based TFTEDs with seven layers, which owned two thermocouples, were also fabricated to contrast with the signal PN junction. Figure 4 shows the open output voltage (V_o_) of the devices as a function of ΔT for the devices. It can be seen from Figure 4 that the output voltage of the seven-layer Ni-based device exhibits a linear increasing trend with the increasing ΔT and the value is about two times that of the device with a single thermocouple as shown in Figure 3, indicating that the multilayer structure devices have good stability. Similarly, the device with a single Bi/Sb PN junction also shows the linear increasing trend with the increasing ΔT. It has a higher output voltage than that of the device fabricated with Ni-based device due to their higher Seebeck coefficient. Assuredly, the Bi_2_Te_3_/Sb_2_Te_3_ based device has the maximum output voltage among the devices. The maximum output voltage increases with the increasing of ΔT and doubles after adding a pair of thermocouples. All of these results suggest that the multilayer structure is promising for preparing high integration devices and applications to exploit various practically available heat sources.

## 3. Conclusions

A novel multilayer structure design is used for preparing a thin-film thermoelectric device. It is shown that the output voltage of all of the prepared thin-film devices have a linear increasing trend with the increasing temperature difference, indicating the reliability of the multilayer structure. The laser sensor test results show that the prepared thin-film device has high sensitivity and a lower response time than that of the commercial laser power detector. Thus, our multilayer structure is a promising new structure that can be used for fabricating high integration and high-performance thin-film devices.

## Figures and Tables

**Table 1:** The detail preparing parameters and properties of the thin films.

	Sputtering Power	Ar Flow	O_2_ Flow	Thickness	Seebeck Coefficient	Electrical Conductivity
NiCu	100 W	40 sccm	-----	370 nm	−28 μV K^−1^	15,300 Sm^−1^
NiCr	90 W	30 sccm	-----	413 nm	15 μV K^−1^	14,000 Sm^−1^
SiO_2_	150 W	30 sccm	5 sccm	563 nm	-----	<0.1 Sm^−1^

**Table 2:** The room-temperature thermoelectric properties of the thin films.

	Seebeck Coefficient	Electrical Conductivity
Sb	47 μV K^−1^	7700 Sm^−1^
Bi	−33 μV K^−1^	6500 Sm^−1^
Sb_2_Te_3_	125 μV K^−1^	4700 Sm^−1^
Bi_2_Te_3_	−97 μV K^−1^	4300 Sm^−1^
